# Baseline T cell dysfunction by single cell network profiling in metastatic breast cancer patients

**DOI:** 10.1186/s40425-019-0633-x

**Published:** 2019-07-11

**Authors:** Silvia C. Formenti, Rachael E. Hawtin, Neha Dixit, Erik Evensen, Percy Lee, Judith D. Goldberg, Xiaochun Li, Claire Vanpouille-Box, Dörthe Schaue, William H. McBride, Sandra Demaria

**Affiliations:** 1 Department of Radiation Oncology, Weill Cornell Medicine, New York, NY 10065 USA; 2 Nodality, 170 Harbor Way, South San Francisco, CA 94080 USA; 3 Department of Radiation oncology, UCLA David Geffen School of Medicine, Los Angeles, CA 90095 USA; 4 Department of Population Health and Environmental Medicine, New York University School of Medicine, New York, NY 10016 USA; 5 Department of Pathology and Laboratory Medicine, Weill Cornell Medicine, New York, NY 10065 USA; 6 Current address: Gilead Sciences, Inc, 303 Velocity Way, Foster City, CA 94404 USA

**Keywords:** Immunotherapy, Radiotherapy, T cell receptor (TCR) signaling, Programmed Death-1 (PD-1)

## Abstract

**Background:**

We previously reported the results of a multicentric prospective randomized trial of chemo-refractory metastatic breast cancer patients testing the efficacy of two doses of TGFβ blockade during radiotherapy. Despite a lack of objective responses to the combination, patients who received a higher dose of TGFβ blocking antibody fresolimumab had a better overall survival when compared to those assigned to lower dose (hazard ratio of 2.73, *p* = 0.039). They also demonstrated an improved peripheral blood mononuclear cell (PBMC) counts and increase in the CD8 central memory pool. We performed additional analysis on residual PBMC using single cell network profiling (SCNP).

**Methods:**

The original trial randomized metastatic breast cancer patients to either 1 or 10 mg/kg of fresolimumab, every 3 weeks for 5 cycles, combined with radiotherapy to a metastatic site at week 1 and 7 (22.5 Gy given in 3 doses of 7.5 Gy). Trial immune monitoring results were previously reported. In 15 patients with available residual blood samples, additional functional studies were performed, and compared with data obtained in parallel from seven healthy female donors (HD): SCNP was applied to analyze T cell receptor (TCR) modulated signaling via CD3 and CD28 crosslinking and measurement of evoked phosphorylation of AKT and ERK in CD4 and CD8 T cell subsets defined by PD-1 expression.

**Results:**

At baseline, a significantly higher level of expression (*p* < 0.05) of PD-L1 was identified in patient monocytes compared to HD. TCR modulation revealed dysfunction of circulating T-cells in patient baseline samples as compared to HD, and this was more pronounced in PD-1^+^ cells. Treatment with radiotherapy and fresolimumab did not resolve this dyfunctional signaling. However, in vitro PD-1 blockade enhanced TCR signaling in patient PD-1^+^ T cells and not in PD-1^-^ T cells or in PD-1^+^ T cells from HD.

**Conclusions:**

Functional T cell analysis suggests that baseline T cell functionality is hampered in metastatic breast cancer patients, at least in part mediated by the PD-1 signaling pathway. These preliminary data support the rationale for investigating the possible beneficial effects of adding PD-1 blockade to improve responses to TGFβ blockade and radiotherapy.

**Trial registration:**

NCT01401062.

## Introduction

Production of transforming growth factor-beta (TGFβ) is increased in breast and other cancers, and elevated TGFβ levels in blood correlate with worse patients outcome [1, 2]. In addition to promoting metastasis and therapy resistance [3, 4] TGFβ has broad immune suppressive activity [5]. Inhibition of TGFβ improved anti-tumor immune responses in pre-clinical studies [6, 7]. Radiation promotes the dissociation of TGFβ from latency-associated peptide (LAP) [8], thus increasing its active form in the tumor microenvironment. We have previously shown in pre-clinical breast cancer models that neutralization of TGFβ was required for the radiotherapy-induced activation of tumor-specific CD8 T cells [9]. The anti-tumor T cells activated by radiotherapy in the presence of TGFβ neutralization were able to cause regression of the irradiated murine tumors and non-irradiated metastases (abscopal effect), resulting in significantly improved survival [9]. However, it was observed that tumor regression in response to treatment with radiotherapy and TGFβ blockade was often followed by recurrence. The recurrence was significantly delayed by addition of anti-PD-1 antibody, indicating that an active PD-1/PD-L1 pathway limited the effectiveness of the treatment [9].

Based on the beneficial effect on survival and tumor regression observed in preclinical studies, we conducted a phase I/II randomized clinical trial to test in advanced, metastatic breast cancer patients the feasibility and efficacy of combining TGFβ blockade by fresolimumab (GC1008), a human IgG4 kappa monoclonal antibody that neutralizes all mammalian isoforms of TGFβ [10], and radiotherapy. Despite a lack of objective or abscopal responses, patients treated with the higher dose of fresolimumab showed a longer median overall survival compared to those of patients receiving the lower dose, suggesting that the treatment could have elicited some degree of anti-tumor immune response, capable of delaying tumor progression. An increase in tumor-specific CD8 T cells during treatment was observed in some patients, supporting this hypothesis [11]. However, in contrast with preclinical models, T cells elicited in patients were clearly unable to cause tumor regression, suggesting a role for other immunosuppressive pathways.

To gain more insights into the mechanisms underlying the clinical findings, we performed functional studies of the circulating T cells from the patients in this trial who had sufficient residual blood specimens, using a multi-parametric technique that enables the quantification of functional immune signaling capacity at a single cell level [12, 13]. When compared to healthy female donors (HD), patients had evidence of T cell dysfunction at baseline that was more pronounced in the PD-1^+^ subset. Our investigation is limited by the small number of residual samples that could be analyzed. However, we were able to obtain preliminary evidence about the relevance of PD-1 pathway in advanced metastatic breast cancer.

## Patients and methods

### Study design and patients

The study design and procedures for the NCT01401062 trial were reported previously [11]. Briefly, patients with stage IV breast cancer that had progressed after at least one course of systemic therapy with at least three distinct metastatic sites were offered participation in a trial testing fresolimumab and radiotherapy. Patients were randomized to either of two doses of fresolimumab (1 versus 10 mg/kg) administered every 3 weeks (weeks 0, 3, 6, 9, 12). A metastatic site was chosen to receive conformal external beam radiation of 3 fractions of 7.5 Gy, to a total of 22.5 Gy, on alternating days over the course of week 1 and 7. The primary endpoint of the study was abscopal response at week 15 based on Immunological Response Criteria (irC) [14]. Results of this trial were recently reported [11].

### Single cell network profiling (SCNP) analysis of peripheral blood lymphocytes

The SCNP assay was performed as described [13, 15] using blood samples from healthy female donors (HD) and patients. The mean age of patients analyzed for TCR signaling was 55.3 (range 35–74) and for healthy donors was 34.4 (range 20–59). We did not prioritize the analysis of a specific T cell subset, but in many cases we did not have sufficient cells in the CD8 T cell compartment to analyze the data and show significance. Briefly, peripheral blood mononuclear cells (PBMC) were thawed and aliquoted at 100,000 cells/well into 96-deep well plates in RPMI 1640 + 10% FBS. After resting for 2 h at 37 °C cells were stained with antibodies directed against PD-1 (clone EH12.1) and PD-L1 (clone MIH1, from BD Biosciences) or were stimulated with anti-CD3-biotin and anti-CD28-biotin (eBioscience) for 15 min, with avidin crosslinking for the last 2 min, to investigate signaling downstream of the T cell receptor (TCR). In some assays cells were pre-treated with 1.5 μg/ml of either the control isotype human IgG4 (Sigma) or pembrolizumab (Merck) for 1 h at 37 °C prior to stimulation. Cells were then fixed with 4% paraformaldehyde, permeabilized with 100% methanol, and stained with a cocktail of fluorochrome-conjugated antibodies recognizing cell surface markers CD3, CD4 (BD Biosciences), CD8, CD14 (Beckman Coulter), and antibodies recognizing intracellular signaling molecules p-ERK (T202/T204), p-AKT (S473). Live cells were gated using antibody against cPARP (Asp214) (Nodality). Flow cytometry data were acquired on a BD FACS Canto system using the FACS DIVA software (BD Biosciences), and analyzed with WinList (Verity House Software, Topsham, ME). Daily QC of the Canto cytometers was performed as previously described [15].

The response to TCR stimulation was measured using p-AKT and p-ERK as a readout. The raw instrument median fluorescence intensities (MFIs) were converted to calibrated intensity metrics, Equivalent Number of Reference Fluorophores (ERFs), by using rainbow calibration particles. The “Fold” metric was applied to measure the level of a signaling molecule in a condition of interest (e.g. modulated state) compared to its level in a reference well (e.g. un-modulated state). A log2Fold value of 1 implies a 2-fold change in ERF intensity whereas a value close to 2 implies a 4-fold change. The “Fold” metric is calculated as follows: log2Fold: log2 [ERF(modulated state)/ERF(un-modulated state)].

### Statistical methods

Distribution of disease characteristics of patients with and without adequate materials for SCNP evaluation were summarized with descriptive statistics and compared between the 1 mg/kg and 10 mg/kg arms using two sided Fisher’s Exact Tests. Baseline characteristics were summarized with descriptive statistics by site and compared between the 1 mg/kg and 10 mg/kg arms for patients with and without immuno modulatory receptors and for patients with and without TCR signaling analysis. The quantitative variables were summarized using means and SDs and medians with ranges for each arm and compared using two-sided two sample T tests. Qualitative variables were summarized by the distributions of the nominal levels and were compared using two sided Fisher’s Exact Tests. No adjustments for multiple testing were used.

PBMC data obtained form SCNP were analyzed using GraphPad Prism software (GraphPad version 6), and *P* values were calculated using unpaired Student’s two-tailed *t*-tests. All reported *p* values are two-sided and statistical significance defined as *p* < 0.05.

## Results

Results of the clinical study were previously reported [11]. Briefly, there were no significant differences in the distributions of the baseline patient characteristics, or the number of infusions received between arm 1 and 2. Patients in arm 2 had significantly lower risk of death compared with arm 1 (HR arm 1 to arm 2: 2.73 with 95% CI: 1.02, 7.30; *p* = 0.039). However, there were no objective abscopal responses, the primary endpoint of the study, suggesting that blocking TGFβ with radiotherapy was not sufficient to elicit an anti-tumor immune response to control systemic tumor growth. We previously reported extensive phenotypic characterization of circulating lymphocytes, revealing an increase in central memory CD8 T cells and decreased myeloid-derived suppressor cells (MDSCs) in the high dose fresolimumab treatment group [11]. However, phenotypic analyses do not provide information about cellular function. To gather more insights into the barriers to clinical response, PBMC were analyzed using SCNP [12, 13].

Residual blood samples were available from 15 out of 22 patients (6 of 11 from arm 1, and 9 of 11 from arm 2), although not all samples at each time point had sufficient cells for each of the analyses performed. PBMC from 7 HD were used for comparison. Based upon demographic and clinical characteristics, the subset of patients for whom sufficient samples were available is representative of the patients in the entire treatment group (Supplementary Table S1-S3).

**Fig. 1:**
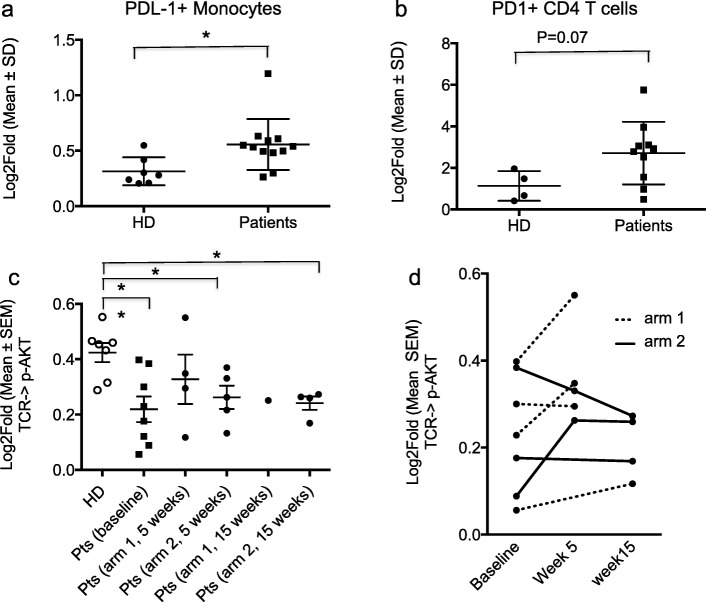
Increased PD-L1 expression and decreased TCR signaling in breast cancer patients PBMC compared to HD. **a-b** Samples were analyzed at baseline for expression of immunomodulatory receptors. **a** Significantly higher levels of PD-L1 on monocytes of breast cancer patients compared to healthy donors (HD). **b** Higher levels of PD-1 on CD4 T cells of breast cancer patients compared to HD. **c, d** TCR modulated signaling in CD8 T cells from HD and patients at baseline and during treatment, as measured by phosphorylation of AKT. **c** Analysis of all available samples from patients treated in arm 1 (1 mg/kg of fresolimumab) and arm 2 (10 mg/kg of fresolimumab) shows overall reduced TCR signaling in CD8 T cells of patients compared to HD. Each dot represents one individual. Unpaired t test, two-tailed, *p < 0.05, **P < 0.005. (D) pAKT values were plotted overtime for 7 patients with baseline and at least one post-treatment sample measurements

For those patients with sufficient samples for SCNP there was an elevated (*p* < 0.05) expression of PD-L1 in patients’ monocytes compared to that in HD (Fig. 1a). Expression of PD-1 on CD4 T cells was also elevated in patient samples compared to HD (*p* = 0.07) (Fig. 1b). Patients randomized to arm 1 or 2 did not differ with respect to the distributions of PD-L1 levels on monocytes (arm 1 vs arm 2, mean 3.10 ± 1.7 vs 2.44 ± 1.13, *p* = 0.53) or PD-1 levels on CD4 T cells (arm 1 vs arm 2, mean 0.51 ± 0.12 vs 0.59 ± 0.29, p = 0.53).

T cell functionality was evaluated by assessing signaling downstream of the T cell receptor (TCR) in samples obtained from subjects during the course of treatment. At baseline, TCR modulated signaling as measured by p-AKT levels was significantly (*p* < 0.005) lower in CD8 T cells from patients compared to HD (Fig. 1c). On a cell population-based analysis, including all samples collected during treatment at weeks 5 and 15 for which TCR modulated signaling could be evaluated, the evoked p-AKT levels remained generally below the levels in HD (Fig. 1c). For a few patients it was possible to compare baseline and at least one post-treatment time point. Some patients showed an increase, others no change or a decrease from baseline in the evoked p-AKT levels. An opposite trend was observed in the only two patients for whom data were obtained for both week 5 and week 15 (Fig. 1d). Overall, this exploratory analysis suggests that treatment with fresolimumab was insufficient to recover T cell functionality in these patients.

**Fig. 2:**
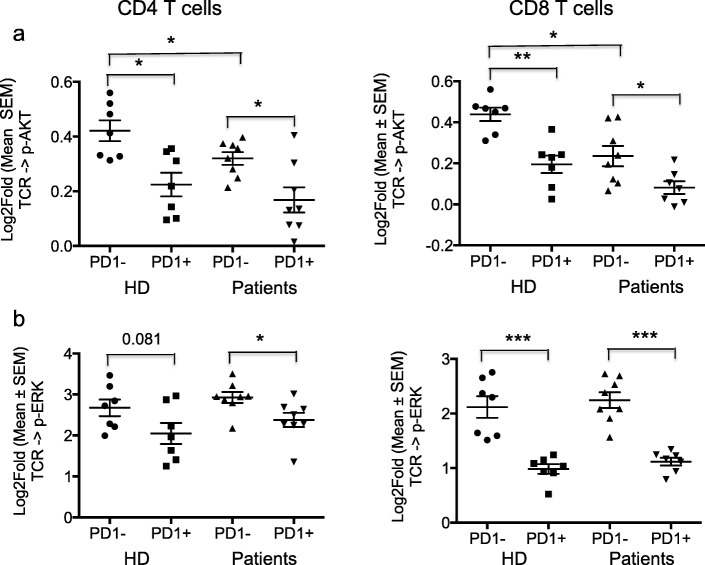
PD-1^+^ CD4 and CD8 T cells show decreased TCR signaling compared to PD-1^-^ T cells. **a, b** The response to TCR stimulation was measured using p-AKT and p-ERK as a readout. PD1^+^ T cells from both HD and patients collected at baseline show significantly reduced TCR modulated signaling compared to PD1^−^ T cells, as measured by phosphorylation of AKT (**a**) and ERK (**b**). Significantly reduced TCR modulated AKT phosphorylation is also evident in PD1^−^ T cells from patients compared to HD. Each dot represents one individual and data is represented as Log2Fold value. A log2Fold value of 1 implies a 2-fold change in Equivalent Number of Reference Fluorophores (ERFs) intensity whereas a value close to 2 implies a 4-fold change. *p < 0.05, **P < 0.005, ***P < 0005

**Fig. 3:**
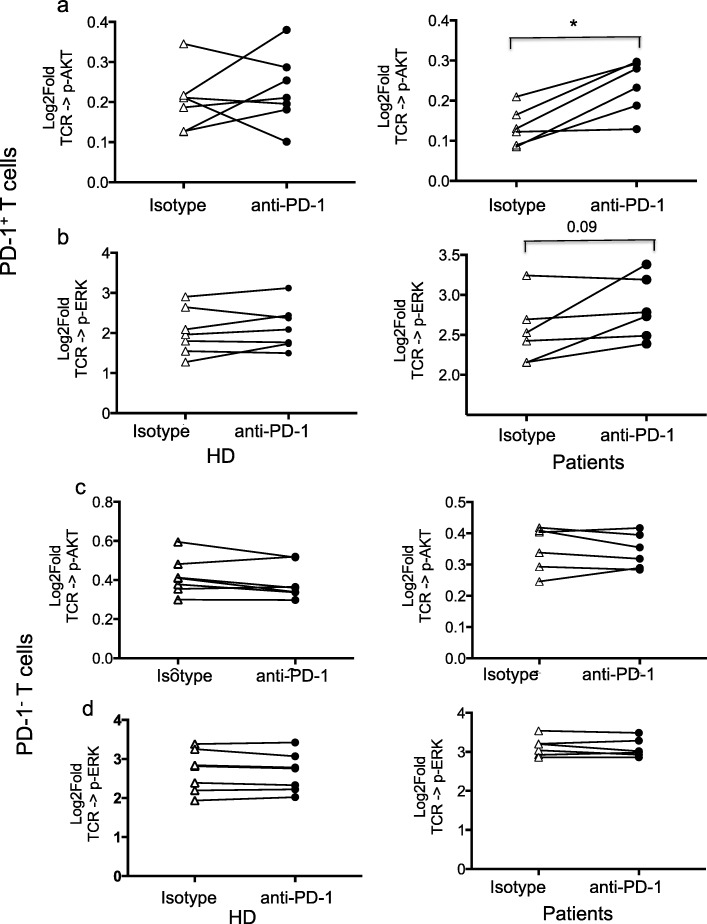
Anti-PD-1 antibody restores TCR signaling in PD-1^+^ CD4 T cells from breast cancer patients. **a-d** PBMC from HD (Left panels) and patients (Right panels) were stimulated with anti-CD3 and anti-CD28 in the presence of anti-PD-1 antibody pembrolizumab (anti-PD-1) or isotype control antibody (Isotype). Graphs show the results for PD1^+^ T cells, as measured by phosphorylation of AKT (**a**) and ERK (**b**). Stimulation in the presence of anti-PD-1 did not significantly increase the response of PD1^+^ CD4 T cells from HD, but increased the response of PD-1^+^ CD4 T cells from patients, achieving significance as measured by AKT phosphorylation and showing a trend to significance, as measured by ERK phosphorylation. Stimulation in the presence of anti-PD-1 did not significantly increase the response of PD1^−^ CD4 T cells from HD and patients as measured by phosphorylation of AKT (**c**) or ERK (**d**). Each line corresponds to one individual. T test, two-tailed, *p < 0.05, **P < 0.005

Further analysis showed that TCR-modulated signaling through p-AKT and p-ERK was consistently and significantly lower in PD-1^+^ as compared to PD-1^−^ T cells, in both CD4 and CD8 T subsets from HD as well as baseline samples from patients (Fig. 2a and b). This was most evident in TCR modulated p-ERK signaling in CD8 cells from patients and HD. To test whether PD-1 blockade in vitro could restore TCR-modulated signaling, PBMC were stimulated in the presence of anti-PD-1 antibody pembrolizumab. PD-1 blockade was associated with increased TCR-modulated signaling through p-AKT and p-ERK in PD-1^+^ CD4 T cells of patients. This effect was not observed in PD-1^−^ T cells (Fig. 3). Insufficient cells were available to perform the same assessment for CD8 T cells.

Overall, these results indicate that the patients enrolled in this study had impaired T cell functionality, and suggest that TCR signaling was not corrected by treatment with fresolimumab and radiotherapy. In vitro PD-1 blockade was able to increase TCR signaling in PD-1^+^ CD4 T cells in baseline samples, suggesting a role for the PD-1 pathway in reduction of TCR signaling through p-AKT and p-ERK in this patient population. Of note, 5 out of the 6 patients tested had hormone receptor-positive (HR+) and 1 had HR + HER2+ breast cancer, suggesting that T cell dysfunction is common in HR+ breast cancer. TCR signaling via p-AKT was reduced, albeit to a lesser degree, also in PD-1^−^ CD4 and CD8 T cells from patients compared to HD (Fig. 2a), and could not be rescued by in vitro pembrolizumab, suggesting a role for other immune checkpoints in the regulation of TCR signaling in these T cell subsets.

## Discussion

In an attempt to describe the immune resistance signature behind the lack of abscopal responses in a phase I/II clinical trial of radiotherapy and fresolimumab in metastatic breast cancer [11] we conducted SCNP exploratory studies. While phenotypic analyses provide information regarding the changes occurring in peripheral blood during cancer treatment, they cannot establish the relationship of these changes with cell subset functionality. To measure the functional capacity of peripheral blood T cells we applied SCNP analysis to patient and HD PBMC, examining TCR modulated signaling via p-AKT and p-ERK in T cell subsets defined by CD4/CD8 and PD-1 expression.

This analysis showed increased baseline levels of PD-1 on CD4 T cells, and PD-L1 on circulating monocytes of breast cancer patient samples, as compared to HD. Importantly, signaling downstream of the TCR was reduced compared to HD in both CD8 and CD4 T cells at baseline, and this was largely unaffected following treatment with fresolimumab. Resolving this analysis to the level of PD-1 expression and association, PD-1^+^ T cells consistently showed lower TCR signaling as compared to PD-1^−^ T cells. Interestingly, in vitro PD-1 blockade with pembrolizumab recovered, at least in part, the ability of PD-1^+^ T cells from patients to signal through these pathways. These hypothesis-generating data provide preliminary evidence that patients with advanced metastatic breast cancer have dysfunctional TCR signaling through p-AKT and p-ERK that is associated with PD-1 expression and that this is detectable in peripheral blood. It remains to be tested if data obtained from PBMC may serve to represent in part the interaction between PD-L1^+^ monocytes and PD-1^+^ T cells that occur in the tumor microenvironment: future studies are warranted to examine such hypotheses using well annotated paired PBMC and tumor samples from breast cancer patients.

While immunotherapy targeting PD-L1 in combination with chemotherapy has shown efficacy in metastatic triple negative breast cancer [16], HR+ breast cancer is poorly responsive to PD-1/PD-L1 blockade [17]. Our data show that PD-1 blockade can improve TCR signaling in T cells of HR+ breast cancer patients (Fig. 3), suggesting that it should be tested as a component of a combination therapy tailored to HR+ breast cancer. A clinical trial designed to test the efficacy of radiotherapy, endocrine therapy with or without CDX-301 and anti-PD-1 (NCT03804944) will open soon.

Comparison of TCR modulated signaling in PD-1^−^ T cells from patients and HD revealed reduced signaling through AKT but not ERK, in patient samples. These data suggest the possibility that PD-1-independent inhibitory pathways are also up-regulated in the immune cells of breast cancer patients. Due to the limited material available for testing we were unable to further investigate this possibility. The use of SCNP and similar assays can be applied to assess ex vivo the functionality of T cells and identify potential actionable targets.

An obvious limitation of our study is the small number of patients with samples available for this analysis. However, these data generated in primary patient samples are supported by additional preclinical analyses. For example, we have previously shown in a mouse model of breast cancer that dual blockade of TGFβ and PD-1 with radiotherapy resulted in reduced tumor recurrence and improved survival compared to radiotherapy and blockade of either TGFβ or PD-1 alone [9]. Additionally, in other tumor types, dual blockade of TGFβ and PD-1 in a mouse model of T cell exclusion induced immune-mediated tumor regression [18] and in a mouse model of colorectal cancer, blockade of TGFβ signaling rendered tumors susceptible to antibodies targeting PD-1/PD-L1 [19]. Together these data serve as the basis for hypothesis testing in future studies.

Recent evidence has highlighted a key role of stromal TGFβ signaling in preventing tumor infiltration by activated T cells [20]. In urothelial cancer patients TGFβ signaling was associated with T cell exclusion and resistance to anti-PD-L1 antibody treatment [18]. In totality, these data, when combined with the clinical findings of the trial [11] encourage additional clinical testing of combined radiotherapy with both TGFβ and PD-1 blockade in breast cancer. A new bifunctional fusion protein targeting PD-L1 and TGFβ, which is undergoing early clinical testing [21], is a promising candidate for this approach. The emerging data in multiple cancers support a role of TGFβ and PD-1 blockade as critical components of a multi-pronged approach to overcome immune resistance of breast cancer.

## Abbreviations

HD: Healthy donors
MDSC: Myeloid-derived suppressor cells
PBMC: Peripheral blood mononuclear cells
PD-1: Programmed death-1
PD-L1: Programmed death-ligand 1
SCNP: Single cell network profiling
TCR: T cell receptor
TGFβ: Transforming growth factor-beta
